# L-Menthol-Loadable Electrospun Fibers of PMVEMA Anhydride for Topical Administration

**DOI:** 10.3390/pharmaceutics13111845

**Published:** 2021-11-03

**Authors:** Amalia Mira, Marta Rubio-Camacho, David Alarcón, Enrique Rodríguez-Cañas, Asia Fernández-Carvajal, Alberto Falco, Ricardo Mallavia

**Affiliations:** Institute of Research Development and Innovation in Biotechnology of Elche (IDiBE), Miguel Hernández University (UMH), 03202 Elche, Spain; a.mira@umh.es (A.M.); marta.rubioc@umh.es (M.R.-C.); dalarcon@umh.es (D.A.); enrique.rodriguezc@umh.es (E.R.-C.); asia.fernandez@umh.es (A.F.-C.)

**Keywords:** PMVEMA, electrospinning, nanofibers, microfibers, menthol, viability, TRPM8

## Abstract

Poly(methyl vinyl ether-*alt*-maleic anhydride) (PMVEMA) of 119 and 139 molecular weights (P119 and P139, respectively) were electrospun to evaluate the resulting fibers as a topical delivery vehicle for (L-)menthol. Thus, electrospinning parameters were optimized for the production of uniform bead-free fibers from 12% *w*/*w* PMVEMA (±2.3% *w*/*w* menthol) solutions, and their morphology and size were characterized by field emission scanning electron microscopy (FESEM). The fibers of P119 (F_119_s) and P139 (F_139_s) showed average diameter sizes of approximately 534 and 664 nm, respectively, when unloaded, and 837 and 1369 nm when loaded with menthol. The morphology of all types of fibers was cylindrical except for F_139_s, which mostly displayed a double-ribbon-like shape. Gas chromatography-mass spectrometry (GC-MS) analysis determined that not only was the menthol encapsulation efficiency higher in F_139_s (92% versus 68% in F_119_s) but also that its stability over time was higher, given that in contrast with F_119_s, no significant losses in encapsulated menthol were detected in the F_139_s after 10 days post-production. Finally, in vitro biological assays showed no significant induction of cytotoxicity for any of the experimental fibers or in the full functionality of the encapsulated menthol, as it achieved equivalent free-menthol levels of activation of its specific receptor, the (human) transient receptor potential cation channel subfamily M (melastatin) member 8 (TRPM8).

## 1. Introduction

The efficacy of any topical treatment depends on the ability of the pharmaceutical formulation to deliver the required drug to the specific target location and thus to penetrate the different skin layers. Many studies describe the difficulty of crossing these barriers by diffusion, which usually reduces the final bioavailability of the drug [1,2,3]. Different carriers have been tested to overcome this limitation, as well as to stabilize compounds of hydrophobic and/or thermolabile nature that may volatilize or degrade by isomerization or oxidation, such as terpenes. In the particular case of this family of compounds, their topical administration for localized skin disorders also pursues avoidance of their unnecessary, and sometimes problematic, systemic distribution resulting from other administration routes. For all of these reasons, several topical delivery systems of terpenes for skin healthcare applications based on nanosystems have been tested, such as nanocapsules, nanoemulsions, nanogels, nanofibers, and liposomes, most of time complexed with cyclodextrins (CD) [4].

The cyclic monoterpene alcohol L-menthol (hereinafter referred to as menthol) is the major constituent of the essential oils of peppermint and corn mint, and it is well known for its beneficial health properties among other characteristics [5,6]. As a result, menthol can be found in a wide range of commercial products, such as pharmaceuticals, cosmetics, pesticides, and oral hygiene products and as a flavoring agent [7,8]. Additionally, as for medicinal purposes, menthol is present in both prescribed and over-the-counter (OTC) medications for the common cold and respiratory conditions, musculoskeletal pain, gastrointestinal disorders, etc. [8]. One of the major effects of menthol is the sensation of coolness produced when it is inhaled, chewed, consumed, or applied to the skin since it is an agonist of the transient receptor potential cation channel subfamily M (melastatin) member 8 (TRPM8) [9,10,11,12].

TRPM8 is a non-selective tetrameric cation channel with high permeability to calcium (Ca^2+^) and sodium (Na^+^) involved in the transmission and modulation of cold sensation when triggered by different physical and chemical stimuli, such as cold temperatures (10–28 °C) [12,13], cooling agents (menthol and ilicin) [14], membrane depolarization [15], and different synthetic molecules [16,17]. This receptor is mainly found in the thermoreceptor sensory neurons of the peripheral nervous system, although they have also been described in other non-neuronal tissues, including skin cells [18]. Apart from TRPM8, menthol can bind other receptors distributed throughout the central nervous system, such as the κ-opioid receptor (KOR) [6] or the γ-aminobutyric acid (GABA) A receptor (GABAAR) [19], leading to analgesia and anti-inflammatory responses, respectively. Nonetheless, the overall final effect induced by menthol depends on its concentration since low doses (≤1%) depress sensory receptors, doses between 1.25% and 16% stimulate sensory receptors, and high concentrations (≥30%) can induce cold pain [20].

Menthol has shown other valuable biological activities, such as antifungal, antibacterial, antipruritic, antitussive, antiseptic, anticancer, antiviral, and fumigant properties [21,22,23]. Moreover, menthol is considered as a penetration enhancer, i.e., it favors the epidermis permeation of bioactive compounds with poor skin diffusion [8,24,25,26,27]. All of these facts, together with its low potential for toxicity in humans [8], make menthol an ideal candidate for topical biomedical applications. However, in order to preserve its beneficial properties, menthol is usually complexed with other molecules, such as CDs. For instance, many works describe the encapsulation of such complexes, instead of free menthol, into electrospun fibers of different polymers, such as poly(vinyl alcohol) (PVA) [28], poly(l-lactic acid) (PLLA) [29], polyethylene oxide (PEO) [30], polystyrene (PS) [31], and poly(methyl methacrylate) (PMMA) [32], or in a coaxial structures based on gelatin and Balangu seed gum [33] or poly(2-ethyl-2-oxazoline) (PEtOX) and silk fibroin [34] as respective core and shell polymers. In this sense, a recent work of Yildiz et al. (2018) [35] stands out by directly electrospinning CD-menthol complexes without using another polymer, enhancing the water solubility and high temperature stability of menthol.

All of these examples of studies encapsulating menthol into electrospun micro/nanofibers are because of their suitability for meeting all those requirements previously mentioned concerning the topical delivery of terpenes [36,37]. Regarding the materials that can be used for this purpose, copolymers of alternating methyl vinyl ether and maleic anhydride allow the construction of different types of drug-loadable nanostructures that meet clinical needs through relatively facile and short protocols [38,39]; however, they have not been tested for this purpose so far. Based on our previous studies on electrospun nanofibers made of polymers of this chemical family [40,41,42], in this work we used poly(methyl vinyl ether-*alt*-maleic anhydride) (PMVEMA) of two molecular weights, 119 (P119) and 139 (P139), to produce fibers (F_119_s and F_139_s, respectively) loaded with menthol. With this end, the electrospinning process was optimized in order to obtain homogenous, bead-free fibers within the micro/nanoscale. Subsequent assays aimed at determining the menthol encapsulation efficiency and the menthol content stability over time. Finally, after analyzing their cytotoxic effect on two different cell lines, the ability to activate the TRPM8 of the menthol loaded into both types of experimental fibers was also tested.

## 2. Materials and Methods

### 2.1. Materials

Two copolymers of PMVEMA (CAS: 9011-16-9) of different molecular weights, P119 (M_w_: 216; M_n_: 80 kg·mol^−1^) and P139 (M_w_: 1080; M_n_: 311 kg·mol^−1^), were supplied by Merck, Sigma-Aldrich (Saint Louise, MO, USA). Double recrystallized menthol (CAS: 2216-51-5) (≥99%) was also purchased from Merck, Sigma-Aldrich. The solvents employed were high-performance-liquid-chromatography (HPLC) grade and consisted of dichloromethane, acetone, ethanol (Merck KGaA, Darmstadt, Germany), and methanol (VWR International, Radnor, PA, USA).

### 2.2. Instrumentation

#### 2.2.1. Electrospinning

The preparation of nanofibers was performed by using the methodology previously described by Mira et al. (2017) [40]. Briefly, the electrospinning process was carried out in a horizontal set-up, including a 2 mL Discardit II syringe (Becton Dickinson, Franklin Lakes, NJ, USA) from which the polymer solution was pumped through a blunt end stainless steel hypodermic needle 316 of 20 gauge (Merck, Sigma-Aldrich) at a flow rate controlled by a KDS 100 infusion pump (KD Scientific, Holliston, MA, USA). The needle and the aluminum foil collector located at a settled distance were connected to a Series FC high voltage source (Glassman High Voltage Inc., High Bridge, NJ, USA). After a refinement process, an electrospinnable solution containing 12% of PMVEMA in acetone, together with 2.3% *w*/*w* menthol for drug-loaded fibers, was selected. Subsequently, the rest of operational parameters were optimized to achieve the most uniform and smallest fibers. It is of note that hereinafter all concentration percentages are given in *w*/*w*.

#### 2.2.2. Optical Microscopy

Samples were electrospun directly onto the aluminum foil collector on which a microscope slide (Deltalab, Barcelona, Spain) was previously placed. The structure of the electrospun nanofibers was observed by using an optical microscope (Microsystems DMI3000B: Leica, Bensheim, Germany) provided with a compact light supply (Leica EL6000), as well as with a digital camera (Leica DFC 3000G). The 40× objective was used to take the images in phase contrast. As for imaging processing, it was performed manually by using the Leica Application Suite AF6000 Module Systems software. By this methodology, preliminary screening of the electrospun nanofibers was carried out.

#### 2.2.3. Scanning Electronic Microscopy

After the optimization of the electrospinning parameters, samples of selected nanofibers arranged onto a microscope slide covered with aluminum foil, were analyzed (US English) in a Shottky type field emission scanning electron microscope (FESEM) Sigma 300 VP model (Carl Zeiss Microscopy GmbH, Oberkochen, Germany) at low kV without coating. From the images obtained for each sample, 100 measurements of nanofiber diameters were taken and analyzed by using ImageJ software (National Institutes of Health, NIH, Bethesda, MD, USA).

#### 2.2.4. Fourier Transform Infrared Spectroscopy (FTIR)

FTIR spectra were obtained by using a Bruker IFS66s spectrometer (Karlsruhe, Germany). Samples were prepared as KBr pellets at room temperature. For each spectrum, there were recorded 256 scans (sample and background) in the range of 4000–400 cm^−1^ and at a resolution of 2 cm^−1^.

#### 2.2.5. Gas Chromatography and Mass Spectrometry (GC-MS)

The menthol content in the fibers was estimated with a gas chromatography-mass spectrometer (GCMS-QP2010 SE) equipped with a quadrupole detector and an automatic sample injector (AOC-20i/s; Shimadzu, Kyoto, Japan). The GC column employed for the assays was an Agilent J&W capillary HP-5MS UI (30 m × 0.25 mm id, 5% diphenyl–95% dimethylpolysiloxane, film thickness 0.25 μm) (Agilent Technologies Inc., Santa Clara, CA, USA). The protocol employed for these analytical assays was based on a previous work described in the literature [43]. In brief, a temperature of 250 °C and 210 °C was used for the injector and detector, respectively. The carrier gas used was helium at a flow rate of 1.5 mL·min^−1^. As for the program, the initial temperature was 40 °C for 2 min. Then, this temperature was increased up to 240 °C at a rate of 10 °C·min^−1^. Finally, the temperature was raised at a rate of 5 °C·min^−1^ and maintained for 5 min at 270 °C. Menthol calibration curves were created for each batch of experiments, with a concentration range of 50–800 µg·mL^−1^. All values corresponded to the area under the curve obtained for each concentration of menthol. In this way, all the curves were preferably adjusted to a quadratic equation correlating the area with concentration, and R^2^ coefficients were greater than 0.99 in all cases (Figure S1).

The analyzed samples (5–10 mg) corresponded to three independent production batches of nanofibers loaded with menthol. Each sample was analyzed just after its preparation and at 3- and 10-days post-production. They were stored uncovered, but protected from light, at room temperature. Immediately prior to their analysis, samples were dissolved in acetone, filtered with 0.2 µm nylon membranes (Millipore, Bedford, MA, USA) and injected at a concentration between the quantifiable ranges of the corresponding calibration curves. In order to check the concentration of each sample before performing the electrospinning process, an aliquot of the initial solution was also analyzed.

### 2.3. Cell Culture

For the in vitro cell assays of this work, human embryonic kidney cells stably expressing hTRPM8 (HEK293-hTRPM8) [44] and the well-known immortalized human keratinocyte cell line HaCaT were used. Both cell lines were maintained in high glucose (4.5 g·L^−1^) Dulbecco’s modified Eagle’s medium (DMEM) supplemented with 10% *v*/*v* fetal bovine serum (FBS), 100 U·mL^−1^ penicillin–streptomycin, and 2 mM l-glutamine (Gibco, Waltham, MA, USA). Cells were grown in 25 cm^2^ flasks at 37 °C in a humidified 5% CO_2_ air atmosphere.

### 2.4. Cytotoxicity Assay

The potential viability changes in HEK293-hTRPM8 and HaCaT cells induced by the electrospun nanofibers were determined by the Thiazolyl Blue Tetrazolium Bromide (MTT) assay, similar to our previous study [40]. For this task, cells at 90–100% confluence, seeded in 96-well plates 48 h before, were treated with different concentrations of each type of nanofiber (i.e., menthol-loaded and unloaded F_119_s and F_139_s) in cell culture media (100 µL per well). Previously, all the fiber samples were dissolved at 6.8% in acetone (Merck, Sigma-Aldrich) to create 75× concentrated sample stocks, which were stored at −80 °C until use. A 75 mM menthol stock solution in acetone was also included. Testing samples consisted of 2-fold dilutions in cell culture media starting from 1× sample stocks. Thus, the menthol calibration curve encompassed a concentration range spanning from 1000 to 7.8 µM, as for the samples of menthol-containing nanofibers, considering full encapsulation efficiencies. There were also included solvent control samples consisting of acetone concentrations equivalent to those in the corresponding sample dilutions. The acetone-dissolved F_139_s partially precipitated in aqueous solvent, i.e., cell culture media, and therefore their cytotoxic effects were only evaluated at the highest concentration of the planned range.

After 24 h of incubation, treatments were replaced with 0.5 mg·mL^−1^ of MTT (from previously prepared 5 mg·mL^−1^ stock solutions in phosphate-buffered saline (PBS) stored at −20 °C) (Merck, Sigma-Aldrich) in fresh cell culture media (100 µL per well) for 2 h. Then, the media was carefully removed, and the colored product formazan was, subsequently, dissolved in an aliquot of 100 µL of dimethyl sulfoxide (DMSO). Absorbance measurements were acquired at 570 nm in an absorbance microplate reader SPECTROstar^®^ Omega (BMG Labtech, Offenburg, Germany). The resulting values were correlated with cell concentration and expressed in percentage relative to the untreated cells (control group). Experiments were carried out in quadruplicate, and the results are expressed as means with standard deviation (SD) of three independent experiments.

### 2.5. TRPM8 Channel Activity Assays

Functional assays were performed in HEK293-hTRPM8 cells. TRPM8 activity was estimated by measuring the variation of intracellular Ca^2+^ concentration elicited by menthol and by using the fluorescent Ca^2+^ indicator Fluo4-NW (Molecular Probes, Invitrogen, Milan, Italy), as described in Bonache et al. (2020) [45]. Briefly, two days before treatment, cells were seeded in opaque 96-well plates with transparent bottom (Corning Incorporated, Corning, NY, USA) at a density of 30,000 cells per well. Then, cells were washed with Ca^2+^-free Hank’s balanced salt solution (HBSS) (in mM: 138.0 NaCl, 5.3 KCl, 1.3 CaCl_2_, 0.5 MgCl-6H_2_O, 0.4 MgSO_4_-7H_2_O, 4.0 NaHCO_3_, 0.4 KH_2_PO_4_, 0.3 Na_2_HPO_4_) at pH 7.4 and incubated for 1 h with 100 µL of the dye-loading solution, consisting of 6 µM Fluo4-NW supplemented with 2.5 mM probenecid in HBSS. Subsequently, plates were placed into a POLARStar (BMG Labtech) plate reader for fluorescence detection to record 16 measurements (excitation at 494 nm and emission at 516 nm). After a 3-cycle baseline recording prior to stimulation, the experimental samples (prepared as described before for the cytotoxicity assays to reach the desired concentrations by adding 1 µL per well) were inoculated between the third and fourth cycle using a multichannel pipette. The fluorescence increase induced by the treatments was quantified by subtracting the fluorescence value at the third cycle to the maximum fluorescence value obtained after the addition of the experimental compounds. All data were normalized to the highest response elicited by 1000 mM menthol. The specificity of the assay was assessed by blocking the TRPM8 activity with 10 µM AMTB, a well-known TRPM8 antagonist [46]. All experiments were performed in triplicate, and results are shown as means with SD (*n* = 3).

### 2.6. Statistical Analysis and Graphics

Data were statistically analyzed by two-way ANOVA corrected with Tukey’s test for multiple comparisons using Prism 7 (GraphPad software, San Diego, CA, USA). Prism 7 was also used for general data analysis and plotting.

## 3. Results

### 3.1. PMVEMA Fibers Encapsulating Menthol

Due to the low solubility in water and the high volatility of menthol [47], we first proceeded by confirming its solubilization in organic solvents, particularly, dichloromethane, ethanol, and acetone. The menthol in these solutions was then identified and quantified by GC-MS (Figure S1). Among the different PMVEMA-derived polymers to employ in electrospinning, due to our previous knowledge, our first attempts focused on the ester derivative, which is soluble and commercially available in ethanol. In this way, menthol-loaded nanofibers were obtained. However, their residual content in ethanol, as is shown in the GC-MS chromatograms (Figure S2), could interact with the menthol receptor TRPM8 [48] and then interfere with the functional biological assays planned in this study. For this reason, we finally opted for the anhydride PMVEMA of two different molecular weights (P119 and P139), which are soluble in acetone. After testing several polymer concentrations, both of these PMVEMA forms yielded uniform linear fibers at 12%—a percentage that was subsequently selected for the further optimization of the electrospinning procedure. Along this line, a concentration of 2.3% of menthol was also chosen in order to ideally reach 16% of this compound in the resulting solvent-free fibers, a moderately high bioactive concentration with no harmful effects [20].

**Figure 1:**
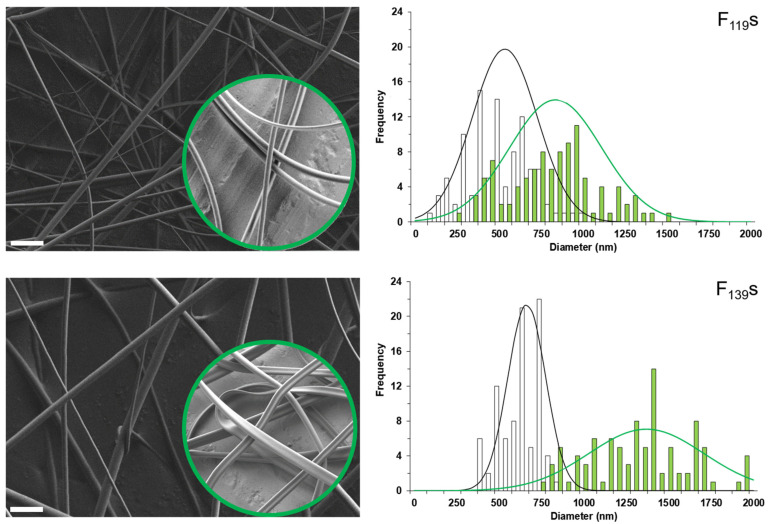
Representative field emission scanning electron microscopy (FESEM) images (**left**) and frequency distribution histograms of the diameter (**right**) of electrospinning-optimized F_119_s and F_139_s. Non-loaded fibers in main images and menthol-loaded ones inside the green-circle insets (scale bars: 5 µm). Each histogram was performed with the data obtained from several images (at least seven) until reaching 100 measurements (white and green bars correspond to non-loaded and menthol-loaded fibers, respectively). Best-fit adjustments to a Gaussian distribution are also correspondingly indicated with a line.

Thus, using the selected concentrations and after testing several experimental conditions, the optimized parameters to produce reproducible uniform fibers without beads were 15.5 kV of voltage, 10 cm of distance between electrodes, and a flow rate of 0.25 mL·h^−1^ (Figure 1).

Assuming cylindrical morphologies, the histogram of the diameter measurements obtained by FESEFM for each type of fiber (100 measurements each) was best-adjusted to a Gaussian distribution with the following parameters (average size ± SD in nm, amplitude, and R^2^, respectively): 524 ± 189, 20.8, and 0.976 for (empty) F_119_s; 837 ± 275, 14.4, and 0.765 for menthol-loaded F_119_s; 664 ± 111, 36.4, and 0.977 for (empty) F_139_s; and 1369 ± 339, 11.8 and 0.788 for menthol-loaded F_139_s. In general, it was observed that the presence of menthol increased the diameter of the fibers, but a further analysis of these histograms also revealed that the non-loaded fibers presented a typical monomodal distribution, while the loaded ones were distributed in two populations. The most obvious explanation for this observation is a heterogenous distribution of the loaded menthol, but in the case of F_139_s, the size of which was the most variable, the addition of menthol showed to profoundly affect their morphology by shaping double ribbon-like structures with distinct sizes depending on the observed dimension (Table S1).

**Figure 2:**
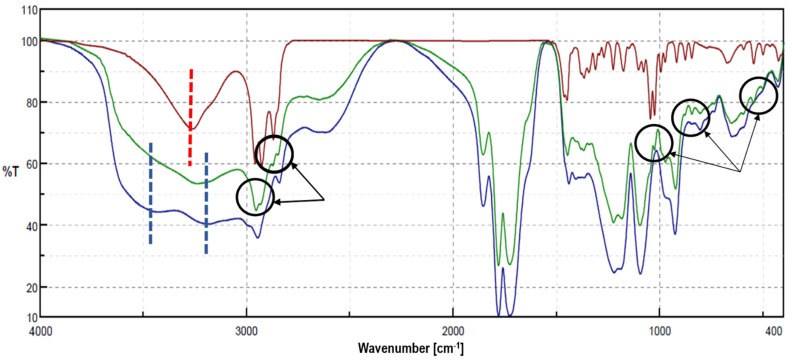
Fourier transform infrared spectroscopy (FTIR) spectra of menthol (red line), F_139_s (blue line), and menthol-loaded F_139_s (green line) in KBr pellets. Evidence of the encapsulated menthol, including the new IR bands, is indicated in circles.

Such an effect on the morphology of the loaded fibers may have been due to powerful Van der Waals interactions, mainly hydrogen bonds between the hydroxide group of menthol and the carbonyl groups of the anhydride. This effect appears to be reflected in the FTIR spectrum of the loaded F_139_s (Figure 2), which shows a slight shift (5 cm^−1^) of the two original anhydride bands (1851 and 1777 cm^−1^), as well as a correspondence with the changes observed between 3300–2500 cm^−1^ (typical OH stretch). The presence of the encapsulated menthol was confirmed by new IR bands in the fingerprint region (1000–500 cm^−1^) and in the saturated alkyl region (less than 3000 cm^−1^).

### 3.2. Determination of the Loaded Content and Its Stability over Time

**Figure 3:**
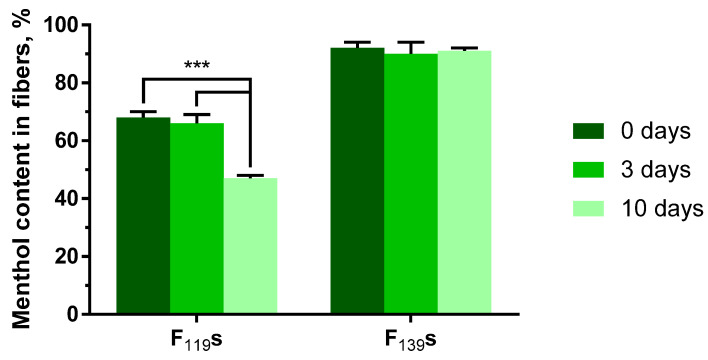
Stability over time of the menthol content in loaded F_119_s and F_139_s. The amount of menthol in acetone-dissolved fibers was analyzed by gas chromatography and mass spectrometry (GC-MS) at 0-, 3- and 10-days post fiber preparation. The data are represented in percentages relative to the amount of menthol initially added. Results are shown as the mean with standard deviation from three independent experiments performed in triplicate. Statistical analysis comprised two-way ANOVA corrected with Tukey’s test. ***, *p* < 0.001.

For determining both the encapsulation efficiency and stability over time of the menthol loaded into the experimental fibers, the integrating compounds were separated, and the menthol was identified and quantified by GC-MS (see Figures S1 and S3 for further details on the calibration data). Thus, the menthol content in both F_119_s and F_139_s was analyzed right after their preparation 0, 3, and 10 days after that (Figure 3). The amount of menthol in the polymeric solutions prior to their electrospinning was also quantified to confirm its presence and as reference value (i.e., 100% of menthol content). By following this procedure, the resulting encapsulation efficiencies were 68 ± 2% and 92 ± 2% for F_119_s and F_139_s, respectively. Regarding the content stability over time, F_119_s showed a significant menthol decrease of about 20% at 10 days post preparation. In contrast, the menthol content in F_139_s remained stable throughout the testing period.

The loss of menthol appeared to be mostly related to the different molecular weights of the polymers employed rather than to differences in the relative surface area of the materials, which was notably higher in F_139_s (Figure 1). The containment stability of F_139_s ribbons could also be due to the above-mentioned molecular interactions between menthol and the maleic anhydride groups of P_139_ (Figure 2), which were more numerous than in P_119_. In addition, the volatile nature of the encapsulated compound contributed negatively to its retention if other forces did not intervene in the other way, as we already observed in a previous work when encapsulating methyl salicylate [41].

### 3.3. Cytotoxicity Induced by Experimental Electrospun Nanofibers In Vitro

The potential toxicity induced in cells when treated with the electrospun fibers of this work was also evaluated. For these assays, HEK293-hTRPM8 and HaCaT cells were incubated for 24 h with different concentrations of the experimental samples previously dissolved in acetone. The concentrations used ranged from 7.8 to 1000 µM of menthol (for its calibration curve and menthol-loaded F_119_ samples), testing equivalent amounts of non-loaded F_119S_ samples.

**Figure 4:**
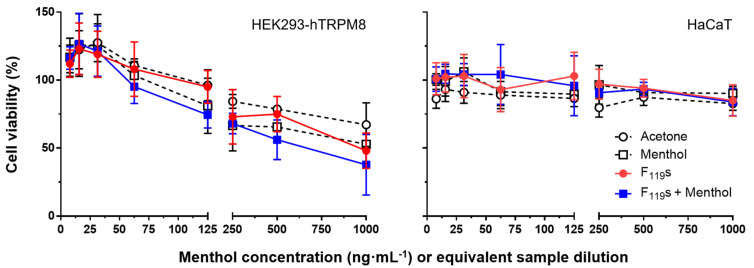
Viability of HEK293-hTRPM8 and HaCaT cells treated with F_119_s experimental samples. Treatments include a gradient of menthol concentrations and equivalent dilutions of F_119_s, menthol-loaded F_119_s, and acetone. Cell viability is calculated in percentage relative to non-treated control cells from the optical densities (570–620 nm) obtained at 24 h post treatment by the Thiazolyl Blue Tetrazolium Bromide (MTT) method. Results are shown as the mean with standard deviation from three independent experiments performed in quadruplicate. Statistical analysis comprised two-way ANOVA corrected with Tukey’s test for multiple comparisons, but no significant differences were found between the treatment groups at each concentration.

As shown in Figure 4, the increase in sample concentration implied a decrease in cell viability in both cell lines. However, no significant differences were found in any of the cell lines between the treatments used, including the solvent control with acetone, suggesting that the observed toxicity was primarily induced by the vehicle (two-way ANOVA, *p* = 0.8763 and *p* = 0.9383 for HEK293-hTRPM8 and HaCaT cells, respectively). Such effect was more obvious in the HEK293-hTRPM8 cell line, which exhibited the lowest cell viability levels for all sample types at the highest dose used (1000 µM, 37.8–67.2%). HaCaT cells were much less sensitive to the cytotoxic effect caused by the treatments, and in none of the cases did the viability levels drop below ~80%. Regarding F_139_s, they were only tested at the highest concentration of the range, displaying comparable results with those of the F_119_s (data not shown).

### 3.4. Assessment of the Activation Capacity of TRPM8 by the Encapsulated Menthol

**Figure 5:**
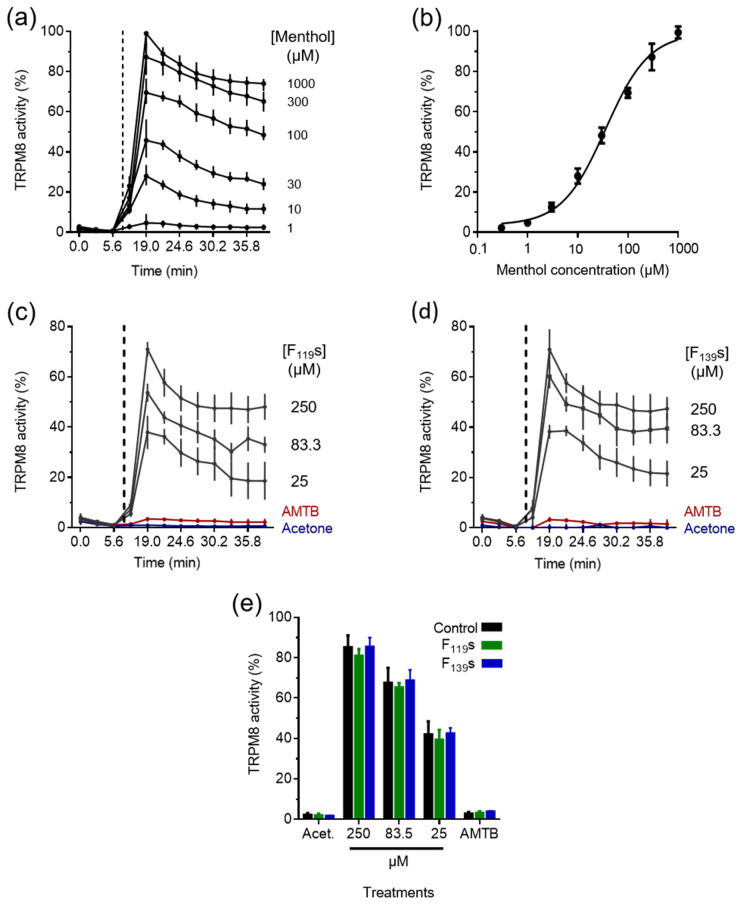
Activation of TRPM8 by the menthol contained in electrospun F_119_s and F_139_s in HEK293-hTRPM8 cells. All data correspond to intracellular Ca^2+^ measurements and are represented as mean percentages with standard deviation of the maximum response observed for each treatment to that of 1000 µM menthol (*n* = 3). (**a**) Representative dynamics of the intracellular Ca^2+^ in response to different menthol concentrations. (**b**) Dose–response curve to menthol. (**c**,**d**) Representative dynamics of the intracellular Ca^2+^ in response to different concentrations of menthol encapsulated in F_119_s and F_139_s, respectively. (**e**) Intracellular Ca^2+^ increases recorded in response to 250, 83.3, and 25 µM of free and encapsulated menthol, including as controls: samples without menthol and 0.05% acetone (Acet.), and the specific TRPM8 blocker AMTB at 10 µM against 83.3 µM menthol samples. Statistical analysis calculated from the datasets in (**e**) comprised two-way ANOVA corrected with Tukey’s test for multiple comparisons, but no significant differences were found between the treatment groups at each concentration.

The menthol encapsulated into the experimental fibers after being dissolved in acetone, as for the cytotoxicity assays, induced intracellular Ca^2+^ increases in a concentration-dependent fashion in treated HEK293-hTRPM8 cells (Figure 5). First, TRPM8 activation dynamics were determined for our experimental model using free menthol (Figure 5a,b), describing a dose–response curve as in previous works [49].

Follow-up studies showed that the menthol contained in both types of fibers was able to activate TRPM8 similarly, and also in a concentration-dependent manner (Figure 5c,d for F_119S_ and F_139S_ samples, respectively). The vehicle (0.05% acetone) did not induce Ca^2+^ internalization (Figure 5c–e), nor did the fiber samples without menthol (data not shown). TRPM8 activation was specific, as the observed Ca^2+^ increase in cells was abolished with the TRPM8 blocker AMTB (Figure 5e). In Figure 5e, the representation of the highest Ca^2+^ internalization values induced by different concentrations of menthol shows equivalent levels of TRPM8 activation between the free and the encapsulated compound, indicating no significant modifications in the loaded menthol after performing the electrospinning process. The concentrations chosen for this assay were 250, 83.3, and 25 µM, which were all comprised within the linear region of the dose–response menthol calibration curve.

## 4. Conclusions

In this work is described a procedure for obtaining uniform, beadless electrospun nano/microfibers loadable with 16% of the bioactive model terpene menthol without using CD, but with two PMVEMA derivates differing only in their molecular weights (F_119_s and F_139_s). All the resulting non-loaded fibers showed uniform, beadles, and cylindrical morphologies. However, when loaded with menthol, such difference in polymer molecular weight affected the obtained fibers by shaping ribbon-like F_139_s endowed with much higher encapsulation efficiency and content stability over time than the cylindrical-loaded F_119_s. Regarding their interaction with cells, none of the two types of fibers showed significant levels of toxicity in HaCaT and HEK293-hTRPM8 cells treated for 24 h in the range of concentrations used. Finally, the full functionality of the menthol encapsulated into both types of fibers was also verified in vitro, as it achieved TRPM8 activation levels equivalent to those of the free (i.e., non-manipulated) menthol.

## Data Availability

The data presented in this study are available on request from the corresponding author.

## Supplementary Materials

The following are available online at https://www.mdpi.com/article/10.3390/pharmaceutics13111845/s1, Figure S1: Procedure to quantify menthol by GC-MS, Figure S2: Representative GC-MS spectra of PMVEMA-ES 25% *w*/*w* and PMVEMA-ES 25% *w*/*w* with L-menthol 16% *w*/*w* fibers, Figure S3: GC-MS chromatograms of the samples corresponding to the stability experiment, Table S1: Data of histograms obtained for optimized fibers with and without menthol.
