# Supplementary material for: L-Menthol-Loadable Electrospun Fibers of PMVEMA Anhydride for Topical Administration
Source: Pharmaceutics. 2021 Nov 3;13(11):1845. doi: 10.3390/pharmaceutics13111845 (PMC8618103; doi:10.3390/pharmaceutics13111845)
Supplement: Supplementary file 1 [file pharmaceutics-13-01845-s001.zip › Supporting information Pharm 2021 rev..docx]

Supporting information

L-Menthol-loadable electrospun fibers of PMEVA anhydride for topical administration

Amalia Mira^1a^, Marta Rubio-Camacho^1a^, David Alarcón^1^, Enrique Rodríguez-Cañas, Asia Fernandez-Carvajal^1^, Alberto Falco^1,^* and Ricardo Mallavia^1,^*.


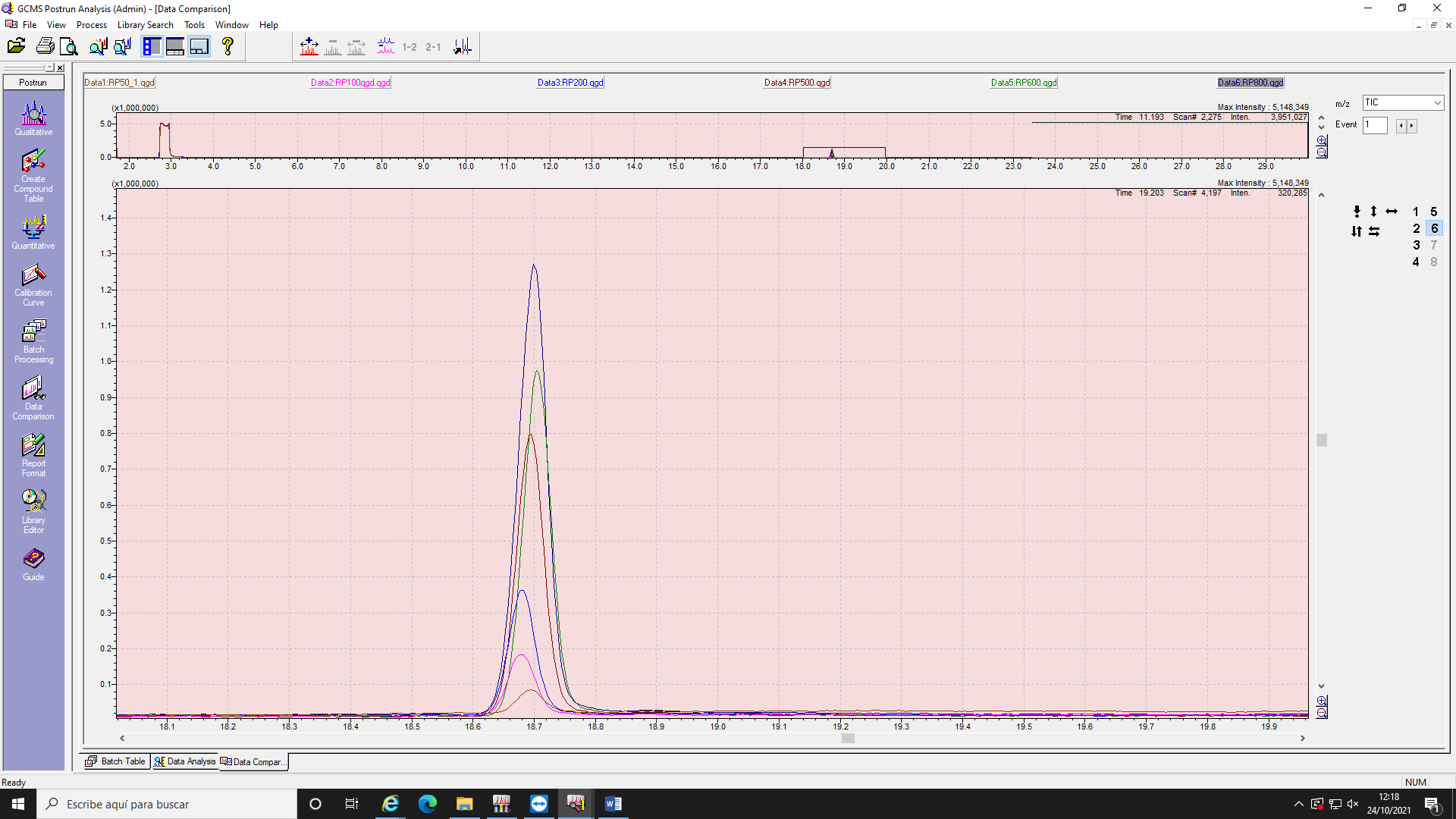

**Figure S1.** Procedure to quantify menthol by GC-MS. Top: Representative chromatograms of the calibration procedure in acetone. Retention time (18.7 min) *versus* intensity signal of menthol. Bottom: Calibration of the integrated area for menthol in three different solvents: dichloromethane (blue), methanol (red), and acetone [1-3].

**Figure S2.** Representative GC-MS spectra of PMVEMA-ES 25% w/w (black) and PMVEMA-ES 25% w/w with L-menthol 16% w/w (pink) fibers. Peaks corresponding to ethanol (1.75 min) and menthol (7.63 min) [1-3].


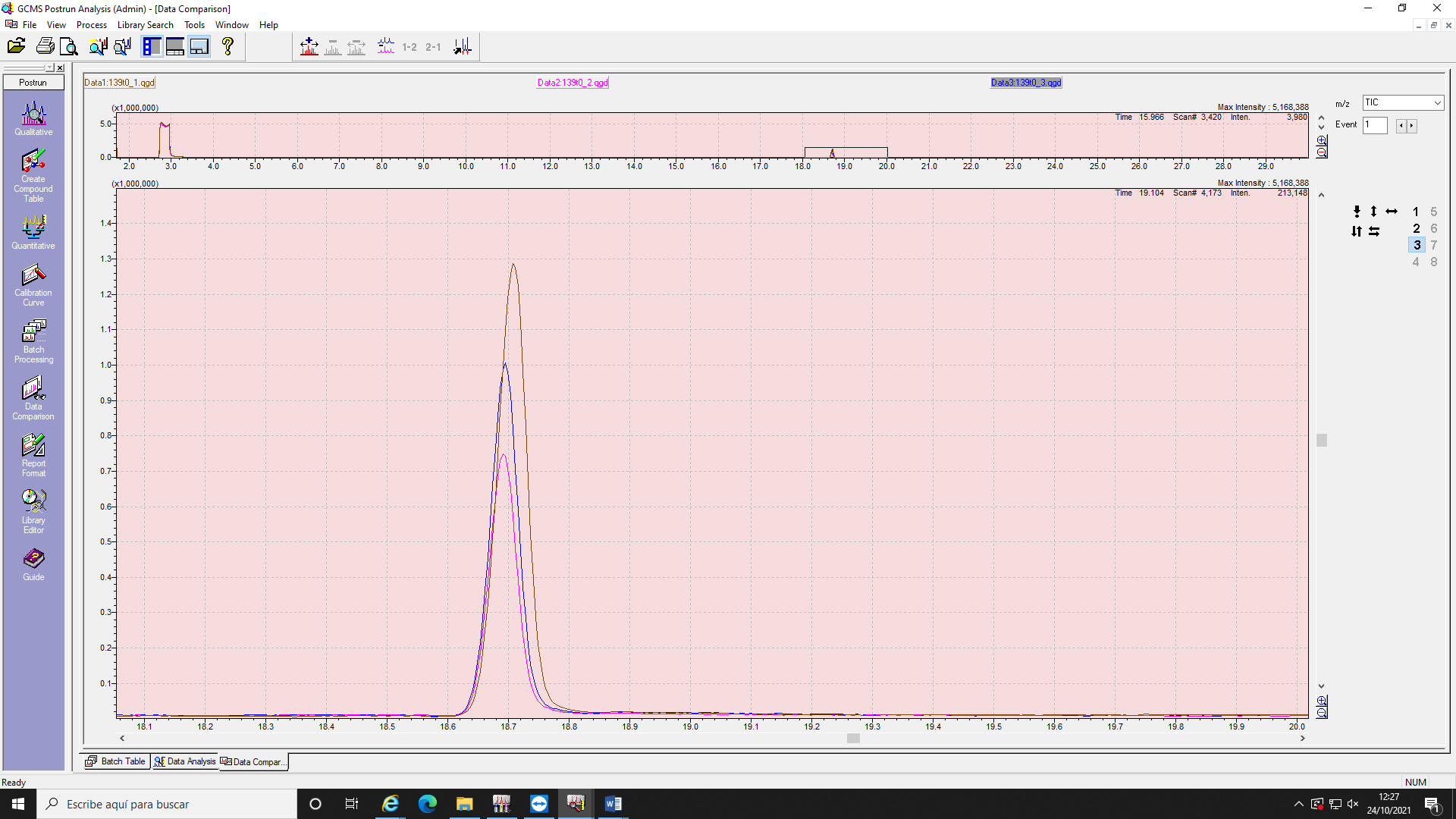

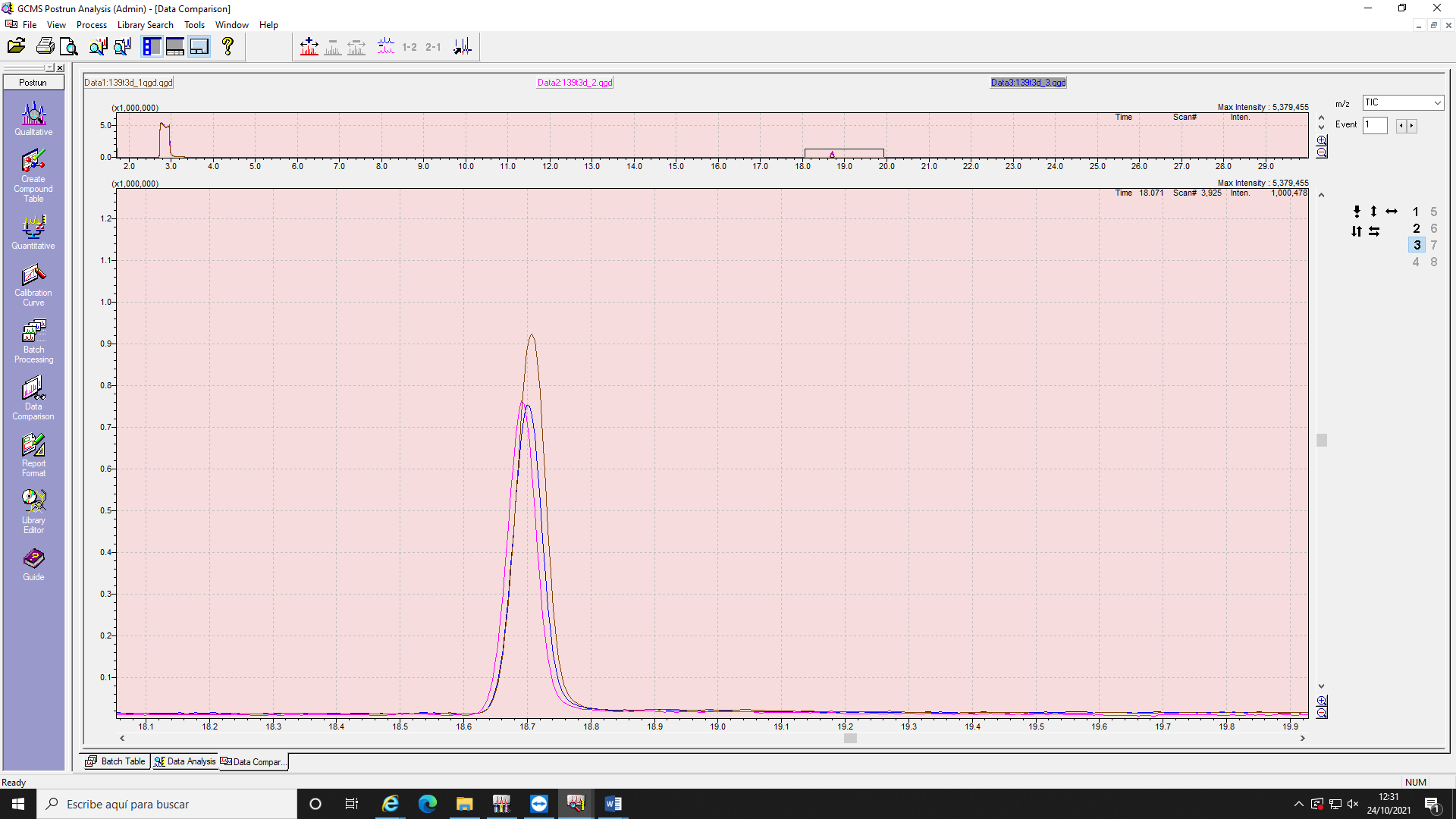

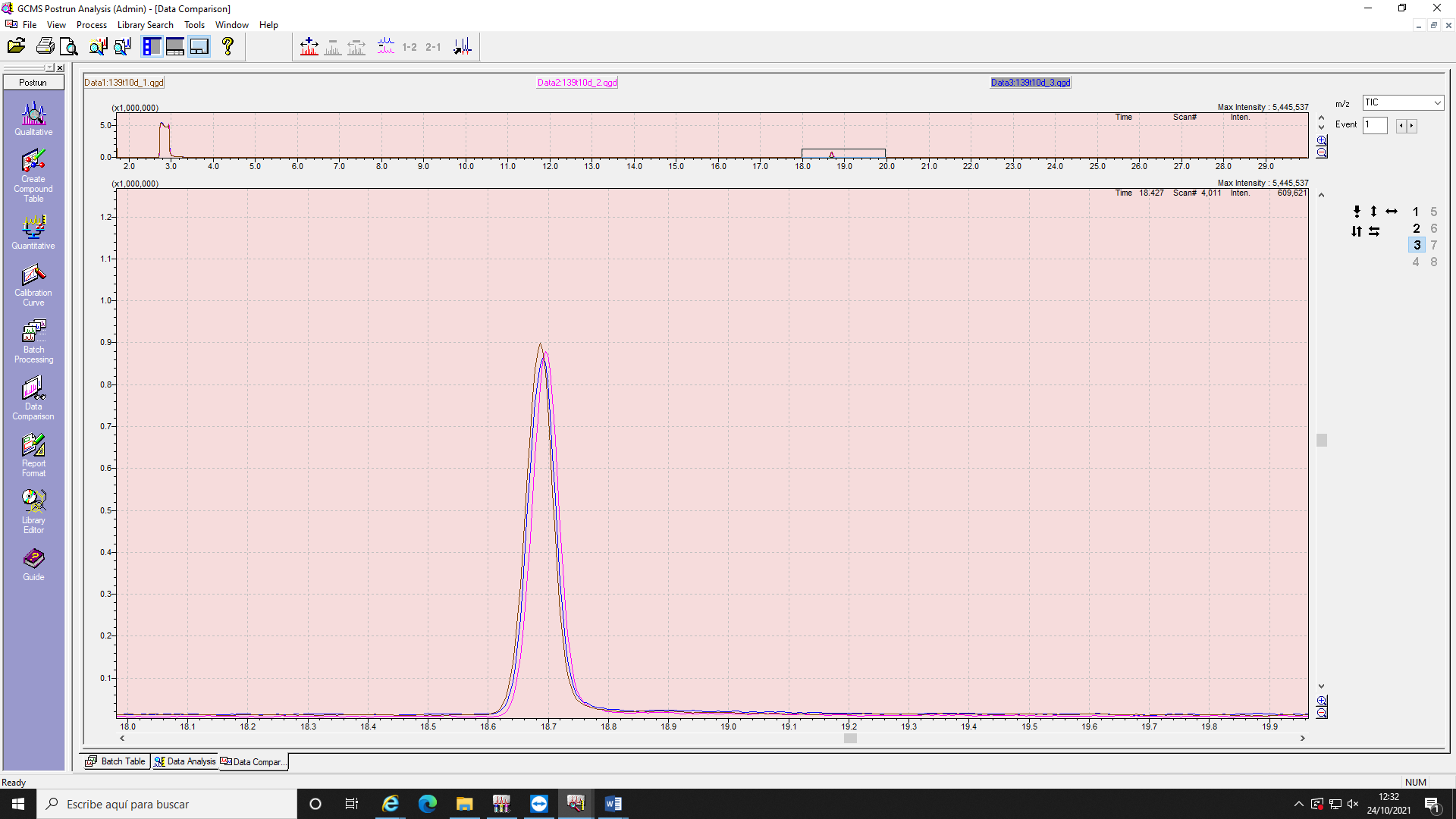


**a**

**b**

**c**


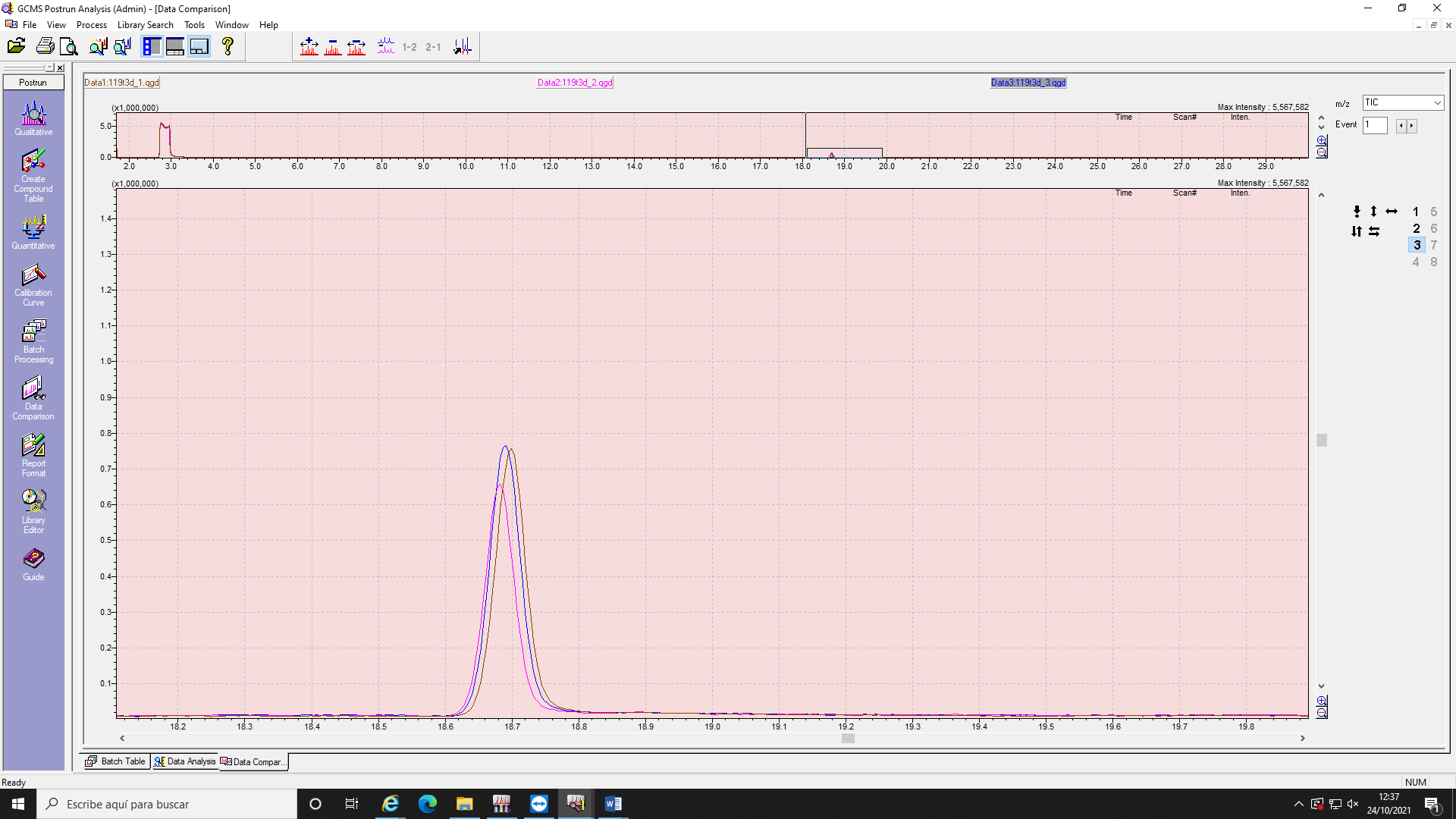

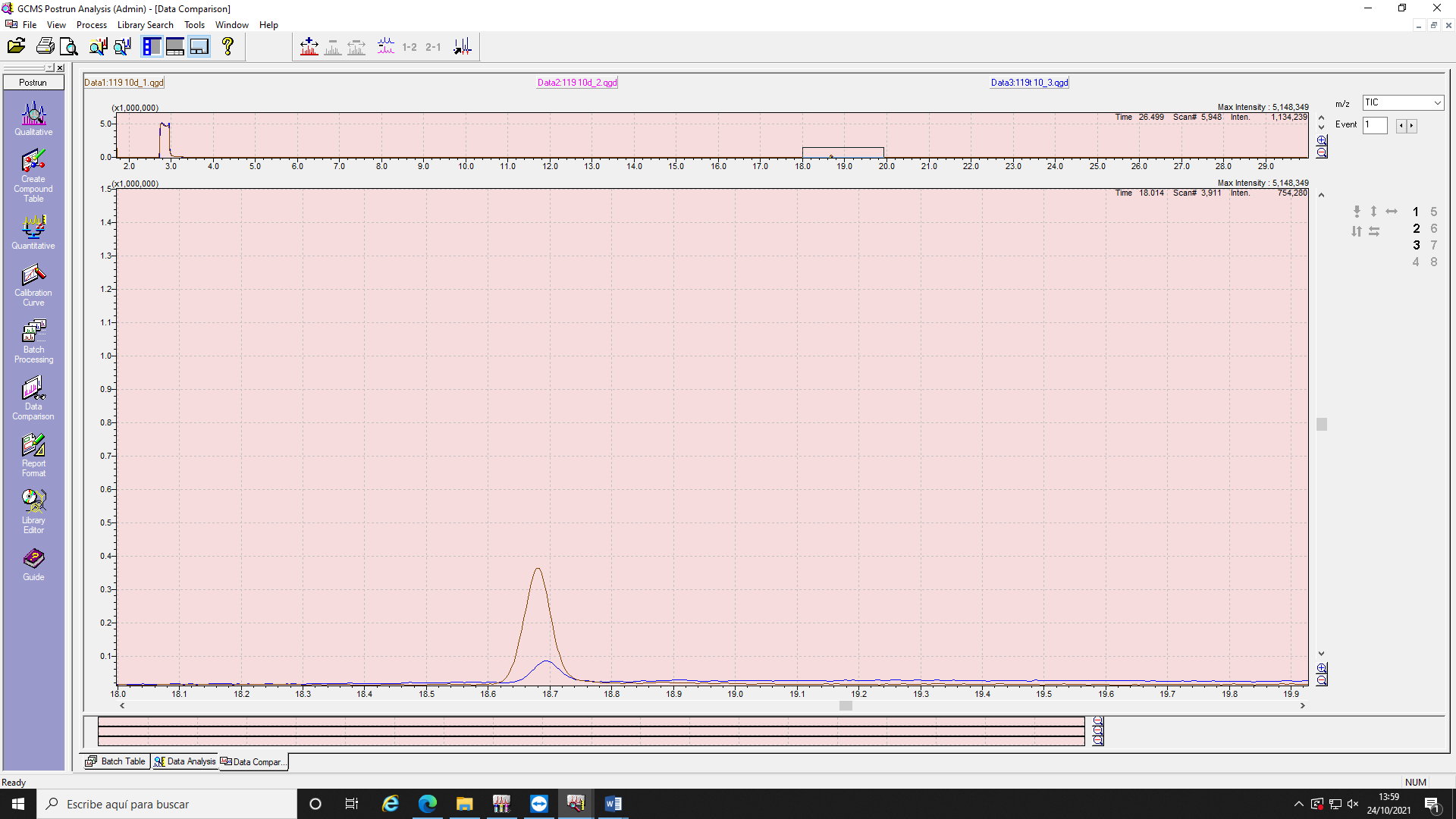

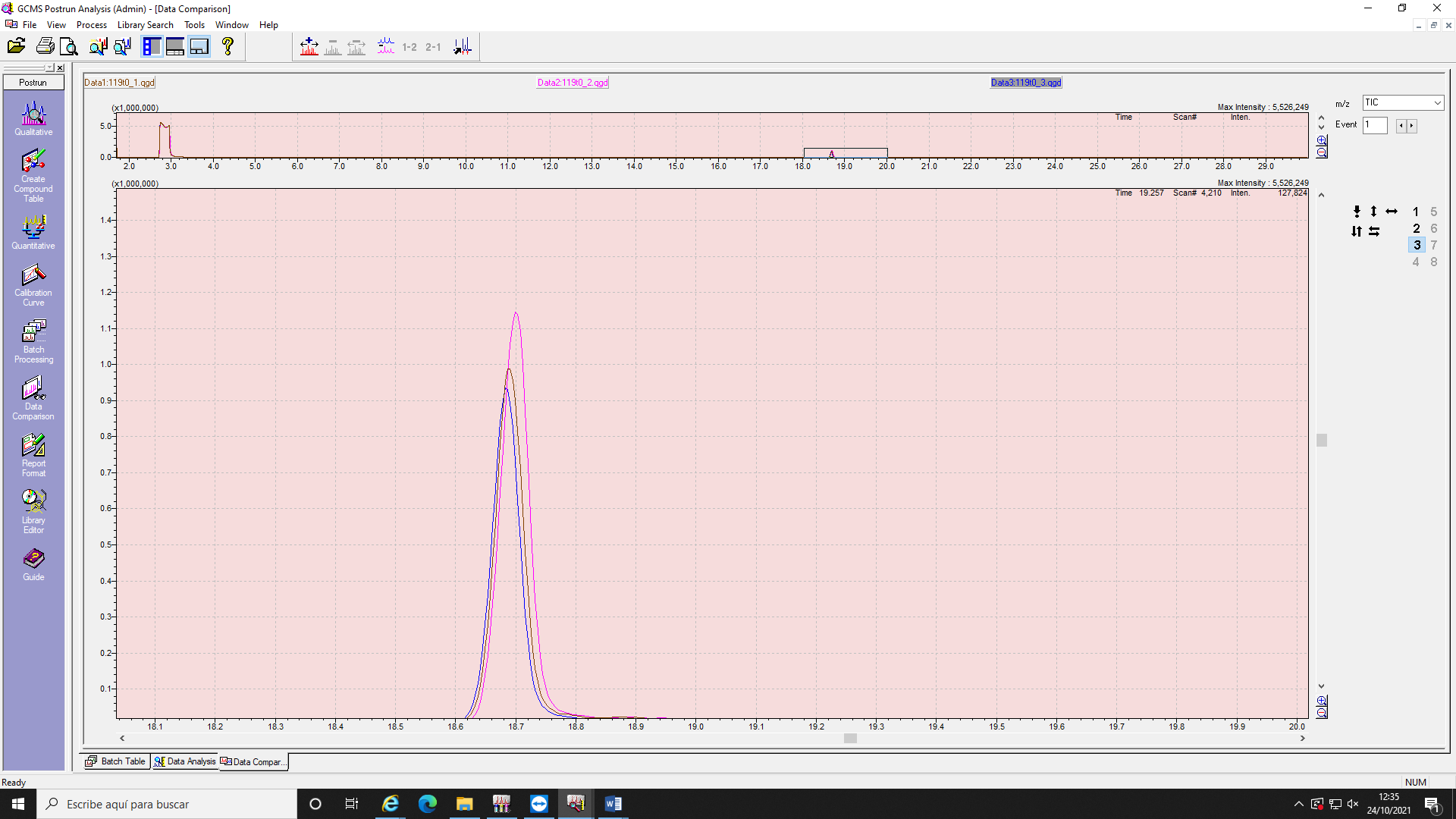


Intensity (u.a.)

Time (min)

**d**

**e**

**f***

**Figure S3.** GC-MS chromatograms of the samples (in triplicate) corresponding to the stability experiment (Figure 3). (a) F_139_s, t=0; (b) F_139_s, t=3d; (c) F_139_s, t=10d; (d) F_119_s, t=0; (e) F_119_s, t=3d; and (f) F_119_s, t=10d (*, two out of three chromatograms overlap here).

**Table S1.** Data of histograms obtained for optimized fibers of two PMVEMA molecular weights without or with L-Menthol at 16% w/w.

| **PMVEMA-Anhydride** | **L-Menthol** | **Diameter (nm)** | **Amplitude (nm)** | **R^2^ *** | **Observations** |
| --- | --- | --- | --- | --- | --- |
| 119 | - | 534±194 | 21 | **0,976** | Monomodal |
|  | + | 837±275 | 14 | 0,765 | Three contributions: empty (16%), **loaded (66%)** and double (18%) |
| 139 | - | 664±111 | 36 | **0,976** | Monomodal |
|  | + | 1369±339 | 12 | 0,788 | Two contributions: loaded (25%) and **double (75%)** |

*95% Confidence intervals

**Bibliography:**

1. Peat J., Frazee, C., Kearns, G and Garg, U.; “Determination of Menthol in Plasma and Urine by Gas Chromatography/Mass Spectrometry (GC/MS)” in Clinical Applications of Mass Spectrometry in Drug Analysis” Methods in Molecular Biology, Springer Protocols, Garg Uttam Editor, (2016), vol. **1383**, cap. 22, p. 205-212. **ISBN 978-1-4939-3252-8 (eBook)**
2. Adib, N.A.M., Mandal, U.K., Mohamed, F. and Chatteriee B.; “Fast and simple gas chromatographic method for simultaneous estimation of camphor, menthol and methyl salicylate in analgesic ointment: application in stability study.” (2017) *Journal of Pharmaceutical Investigation* **47**, 275–285. [**DOI: 10.1007/s40005-017-0305-02**](https://doi.org/10.1007/s40005-017-0305-02).
3. Pérez, J.J., Watson, C.H., Blount, B.C., Valentín-Blasini, L.; “Isotope-Dilution Gas Chromatography-Mass Spectrometry Method for the Selective Detection of Nicotine and Menthol in E-Cigarette, or Vaping, Product Liquids and Aerosols (2021) *Frontiers in Chemistry*, **9**. 754096. **DOI: 10.3389/fchem.2021.754096**.
